# Re-Adaption on Earth after Spaceflights Affects the Mouse Liver Proteome

**DOI:** 10.3390/ijms18081763

**Published:** 2017-08-12

**Authors:** Viktoria Anselm, Svetlana Novikova, Victor Zgoda

**Affiliations:** 1 Interfaculty Institute of Biochemistry (IFIB), Hoppe-Seyler-Straße 4, Tuebingen 72076, Germany; mail@v-anselm.de; 2 Orekhovich Institute of Biomedical Chemistry of Russian Academy of Medical Sciences, Pogodinskaya 10, Moscow 119121, Russia; novikova.s.e3101@gmail.com

**Keywords:** spaceflight, mouse, liver, proteome, metabolism, cytochrome P450

## Abstract

Harsh environmental conditions including microgravity and radiation during prolonged spaceflights are known to alter hepatic metabolism. Our studies have focused on the analysis of possible changes in metabolic pathways in the livers of mice from spaceflight project “Bion-M 1”. Mice experienced 30 days of spaceflight with and without an additional re-adaption period of seven days compared to control mice on Earth. To investigate mice livers we have performed proteomic profiling utilizing shotgun mass spectrometry followed by label-free quantification. Proteomic data analysis provided 12,206 unique peptides and 1086 identified proteins. Label-free quantification using MaxQuant software followed by multiple sample statistical testing (ANOVA) revealed 218 up-regulated and 224 down-regulated proteins in the post-flight compared to the other groups. Proteins related to amino acid metabolism showed higher levels after re-adaption, which may indicate higher rates of gluconeogenesis. Members of the peroxisome proliferator-activated receptor pathway reconstitute their level after seven days based on a decreased level in comparison with the flight group, which indicates diminished liver lipotoxicity. Moreover, bile acid secretion may regenerate on Earth due to reconstitution of related transmembrane proteins and CYP superfamily proteins elevated levels seven days after the spaceflight. Thus, our study demonstrates reconstitution of pharmacological response and decreased liver lipotoxicity within seven days, whereas glucose uptake should be monitored due to alterations in gluconeogenesis.

## 1. Introduction

Spaceflights provide a unique opportunity to study microgravity-related changes in organs on a biochemical level. In 2013, the Institute of Biomedical Problems of the Russian Academy of Sciences (IBMP RAS) targeted this research field with the project “Bion-M1” by sending mice in bio-satellites to the Earth orbit in a 30-days flight [1]. As a metabolically highly active organ, the liver of these mice is particularly interesting for us to investigate the effects of spaceflights. Open questions on the proteome dynamics during prolonged spaceflights and their effect on this vital organ involved in metabolism is of great clinical interest in terms of therapy and medical monitoring. Liver function involves a number of enzymatic systems. Among them, members of the cytochrome P450 superfamily crucial for hepatic drug metabolism are the well characterized [2]. The majority of proteins belonging to the protein families CYP1, CYP2, and CYP3 are involved in metabolism of drugs and xenobiotics with substrate specificity intrinsic to the each particular subfamily [3]. As the level of these CYP enzymes determines the overall drug dosage and therapeutic success, their level and activity should be examined to provide proper medication during prolonged spaceflight and re-adaptation on Earth. Furthermore, the systems controlling carbohydrates, proteins, and lipid metabolism need to be studied in the context of spaceflights in order to develop complete nutrition. Effects of 13-day spaceflights on liver function were previously observed including loss of retinol and activation of peroxisome proliferator-activated receptor (PPAR) pathways indicating early signs of non-alcoholic fatty liver disease (NAFLD) [4]. Quantitative mass-spectrometry using stable isotope labeling or label-free approach represents thereby a powerful method to reveal differentially expressed proteins [5]. Thus, using mass-spectrometry, it was shown that PPAR pathways are crucial for lipid metabolism in the liver [6].

To extend our knowledge concerning liver proteins affected by microgravity, we tried to give insight into their expression levels during re-adaption on Earth. Here we present an approach for proteome profiling of mice livers using shotgun mass spectrometry coupled with label-free quantification as a powerful method to reveal a broad range of differentially expressed proteins. Moreover, functional analysis along clusters of differentially expressed proteins provides a comprehensive overview of the metabolic response of a mammalian system to environmental changes related to spaceflights.

## 2. Results

**Figure 1:**
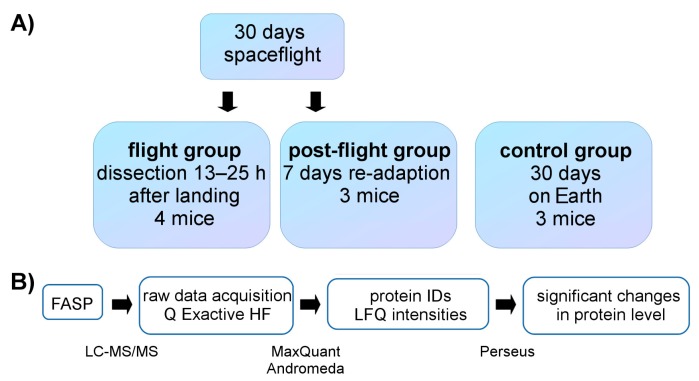
Sample overview and methodological workflow. (**a**) Sample overview for all three groups investigated. The control group on Earth was held in housing and climate conditions corresponding to the conditions of flight group; (**b**) Workflow of sample preparation, processing, and analysis. IDs—identifications; FASP—filter-aided sample preparation; LFQ—label-free quantification.

We studied the effects of microgravity on mice livers by comparing the proteome of mice livers from a 30-day spaceflight, seven day re-adapted mice, and control mice (Figure 1A). Control group animals were housed in the same environmental conditions (e.g., temperature, humidity, gas composition) as in the spaceflight [1]. Three biological replicates were provided for the re-adaption (post-flight) group as well as for the control group, and four replicates were provided for the spaceflight group (flight). The sample homogenates were analyzed by LC-MS/MS using a nano-flow HPLC system and Q Exactive HF mass spectrometer followed by data processing (Figure 1B). Raw data analysis with MaxQuant and statistical data analysis with Perseus provided 12,206 unique peptides and 1086 identified proteins (Table S1), therefrom 1046 proteins were used for relative protein quantification (Table S2) based on at least three unique peptides.

**Figure 2:**
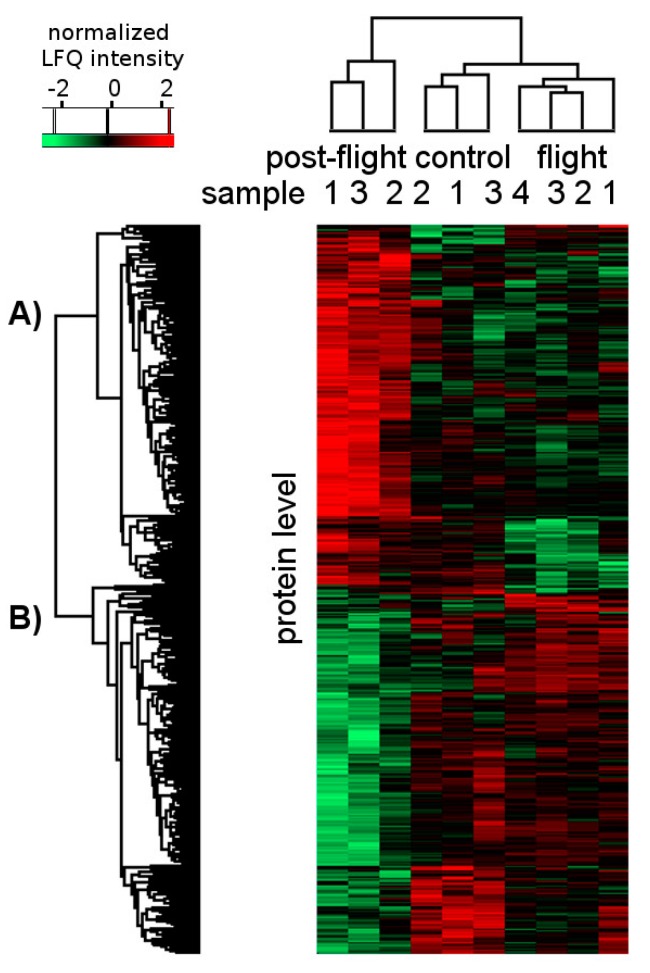
Hierarchical clustering of all proteins with a significantly changed expression profile between at least two groups. Significance was calculated using multiple-sample test (ANOVA model, FDR = 0.05, S0 = 0.1). Samples are clustered in columns and proteins are clustered in rows. Red marked proteins are significantly up-regulated, black marked proteins show no abundance change, and proteins presented in green are down-regulated. Clusters of proteins, which are significantly (**A**) more abundant or (**B**) less abundant in the post-flight group than in the flight or control group are shown. LFQ—Label-free quantification; FDR—false discovery rate

The initially observed label-free quantification (LFQ) intensities were used to examine significant changes in the protein levels between flight, post-flight and control condition by applying an ANOVA test. Quantified proteins of the control group were compared to their levels in the flight group as well as in the post-flight group using unsupervised hierarchical clustering (Figure 2).

Multiple sample testing (ANOVA test, false discovery rate (FDR) = 0.05) resulted in 442 proteins with significantly changed abundances (Table S3). Proteins with significant expression differences are grouped mainly in two large clusters. These clusters comprise 218 (cluster A) and 224 (cluster B) proteins that are up- and down-regulated in the post-flight compared to the other groups, respectively. Furthermore, we observed clustering of three separate sample groups between flight, post-flight, and control condition.

We analyzed all affected proteins regarding their actual function against GeneOntology and Kyoto Encyclopedia of Genes and Genomes (KEGG) databases. Possible clusters of the significant proteins were surveyed by unsupervised hierarchical clustering considering their function, biological process, or cellular component based on ANOVA multiple-sample testing (FDR < 0.05; Figure 2, Table S4). Top 10 annotation terms are represented in Table 1.

In particular, various terms related to ribonucleoproteins or proteins involved in nitrogen compound metabolic processes are enriched in “A” cluster among terms with the highest FDRs (Table 1, Table S4). Additionally, numerous proteins related to amino acid metabolism, especially associated with amine catabolic processes, are part of the list with enriched proteins. The observed protein catabolism activation indicates enhanced gluconeogenesis since degradation protein products serve as carbon substrates during gluconeogenesis [7]. Interestingly, the second protein cluster (Figure 2B) comprising proteins down-regulated in the post-flight group (*n* = 224), is represented by mostly membrane components (Table 1, Table S4). Proteins assigned with terms related to membrane compartments are represented among terms with the highest FDRs in this cluster.

Volcano plots visualizing significance versus fold-change were obtained for control/flight, control/post-flight and flight/post-flight pairs (Figure S1). Applying permutation-based FDR calculation (FDR = 0.05, S0 = 0.1) [8], we determined differentially expressed proteins (Figure S1). Further investigation of significantly differentially expressed proteins (ANOVA test, FDR = 0.05) against KEGG database revealed protein groups of bile secretion, PPAR signaling pathway, retinol metabolism, and cytochromes superfamily (Table S4, Table S5, Table 2).

The proteins associated with bile secretion are less represented with a FDR of 0.019 (Table S4). The level of 10 quantified and bile secretion related proteins (Table 2) is significantly lower in post-flight than in the flight or control group (ANOVA test, FDR = 0.05). The majority of these proteins have ion or cation transporter activity to regulate the bile flow from liver to gall bladder by building an osmotic gradient [9]. A decreased abundance of these transporters in the post-flight group may indicate less bile secretion leading to an accumulation of harmful bile acids in hepatocytes [10]. A major part of our observed bile secretion related proteins is only significantly down-regulated in the post-flight group compared to the flight group (Table 2) which indicates considerable reconstitution of bile secretion within seven days. Notably, there was no difference in the abundance of these transmembrane proteins between the control and flight group, which could be owed to less sensitive fold change detection between flight and control. This could be caused by the high significance threshold observed with multiple testing correction using permutation based FDR (Figure S1, A). Comparisons between control and post-flight or flight and post-flight demonstrate considerable lower significance thresholds. 

The PPAR signaling pathway members are mostly up-regulated. The most prominent level increased (fold change >2) in flight group comparing with post-flight group was found for cytochrome P450 4A12A (Cyp4a12) involved in fatty acid ω-oxidation, fatty acid-binding protein (Fabp1) used for fatty acid transport through blood stream, and CD36 antigen (Cd36) acting as fatty acid translocase with lipoprotein binding function [11]. In contrast, long-chain specific acyl-CoA dehydrogenase (Acadl), medium-chain specific acyl-CoA dehydrogenase, (Acadm) and peroxisomal bifunctional enzyme (Ehhadh) essential for fatty acid β-oxidation in mitochondria and peroxisomes are down-regulated.

Regarding spaceflights induced lipotoxicity [4], our examinations give further insights into the reconstitution of liver metabolism. Increased expression levels of the liver residing fatty acid binding protein (Fabp1, Q3V2F7) indicate an active PPAR pathway [12] which, in turn, initiates adipose cell differentiation ultimately resulting in NAFLD [13]. Fabp1, as well as other lipid binding protein levels are significantly increased in the flight group compared to the post-flight group (Table 2) suggesting an activation of PPAR. The observed significant change diminishes comparing control mice and re-adapted mice.

Differentially expressed proteins are enriched for proteins related toretinol metabolism. Different UDP-glucuronosyl transferases, Cyp1a2, and dehydrogenase/reductase SDR family member 4 (Dhrs4) metabolizing retinoids are up-regulated, whereas Cyp3a13 level is decreased in flight group compared with re-adapted mice (Table 2). Retinoic acids are mobilized in hepatic stellate cells as part of retinol metabolism [14] and act as upstream PPAR activators [15,16]. UDP-glucuronosyl transferases perform glucuronidation of retinoic acids for their solubilization [17,18] followed by secretion. After secretion, retinoic acids activate PPARs in hepatocytes.

Apart from retinol metabolism and PPAR pathway, various members of the cytochrome P450 superfamily were detected to have a significantly changed abundance in the post-flight group. We observed up-regulation of CYP2D subfamily members (Cyp2d9, Cyp2d10, and Cyp2d26), as well as increased level of Cyp4v3, Cyp1a2, Cyp2f2, and Cyp4a12a. Members of CYP3A subfamily (Cyp3a11 and Cyp3a13) were down-regulated. Cyp4a12a showed a five-fold (*p*-value = 0.0113) increased level in the flight group compared to the post-flight group. CYP4A subfamily members are involved in fatty acid ω-oxidation in microsomes [19]. Reconstitution of Cytochrome P450 1A2 was confirmed since there is no significant change between control and post-flight along with detected fold change between flight and post-flight (Table 2). The subfamily CYP2D appears 6.1-(*p*-value = 0.0117) and 2.1-fold (*p*-value = 0.0134) elevated in the flight group compared to post-flight group (Table 2). Repeatedly, no significant difference in cytochromes profile between control and post-flight shows that re-adaption to the usual drug metabolizing CYP levels occurs within seven days after landing. 

## 3. Discussion

Manned spaceflights are promising ways to shed light on basic questions regarding origins of life and the human future. To enable this, questions about the impact of environmental conditions during spaceflights can be addressed testing model organisms such as mice. One of these questions is how long-term adaption to spaceflight environment and re-adaption to Earth is reflected in the liver proteome. Our study design was established to exclude housing effects, which are based on the same environmental conditions concerning temperature, humidity, feeding and gas compositions for all animals studied. We performed label-free proteomic profiling and revealed no significant differences between the proteomes of flight and control group due to insufficient fold change detection combined with high variances within groups. Probably, small sample size contributes to the low significance. 

We have revealed significant differential protein expression between flight and post-flight groups. Interestingly, up- and down-regulated proteins are linked with fatty acid oxidation. Remarkably, we revealed up-regulated proteins involved in fatty acid transport (Fabp1), cell uptake (Cd36), translocation into mitochondria (carnitine O-palmitoyltransferase 2, mitochondrial, Cpt), and activation for β-oxidation (long-chain-fatty-acid-CoA ligase 1 (Acsl1)). Moreover, Cyp4a12 related to fatty acid ω-oxidation is up-regulated whereas enzymes essential for β-oxidation both in mitochondria (Acadl and Acadm) and peroxisomes (Ehhadh) are down-regulated. These results could indicate impaired fatty acid β-oxidation, which could lead to fatty acid accumulation, in turn, possibly causing lipotoxicity that underlies NAFLD pathogenesis. Yet, we should interpret these results with caution since a small sample size and label-free approach applied. Notably, the small sample size is linked with the fact that only 16 out of 45 mice (36%) survived the spaceflight and samples were distributed among large number of scientific teams [1]. Nevertheless, project “Bion-M1” provided unique samples to investigate spaceflight effects on living beings.

Functional annotation against KEGG database revealed PPAR and retinol metabolism signaling pathways, which are enriched by down-regulated proteins in the post-flight group compared with the flight group. This result indicates re-adaption occurs partially within seven days associated with diminishing liver lipotoxicity, which is consistent with the data of Jonscher et al. [4]. Jonscher et al. linked matabolome and transcriptome profile including modulated PPARα pathways with lipotoxicity and NAFLD-like phenotype. Analysis of PPAR α, δ, and γ mRNA level of the samples performed in our laboratory did not show significant level change between the samples (data not shown). However, it has been shown previously that activity of PPARs and its pathway members is regulated by posttranslational modifications including phosphorylation [20]. Further validation of PPAR signaling pathway by the targeted proteomics or PTM analysis will help to obtain direct evidences for the PPAR pathway involvement in re-adaptation after space flight.

Moreover, we observed down-regulated fatty acid binding proteins and UDP-glucuronosyl transferases that can contribute to NAFLD recovery. Since NAFLD leads to severe complications such as increased risk of cirrhosis, type 2 diabetes, and cardiovascular complications [21] it is highly important to maintain a hypocaloric diet both during spaceflight and re-adaption to Earth.

Strikingly, enhanced expression of the CYP4A subfamily, that is responsible for ω-oxidation of fatty acids [4], is induced by the PPAR pathway, which coincides with the observed activation of the PPAR pathway. Additionally, our observation can be supported by a previously observed increase in ω-oxidation products in mice livers which experienced a 13-day spaceflight [4]. However, we detected increased expression of Cyp4a12a in flight compared to post-flight, which is consistent with the previously observed increased amount of ω-oxidation products in the flight group [4]. The cytochrome P450 family member Cyp4a12a should be investigated further regarding its amount to confirm an increased level in the flight group and to be measured during different time periods for re-adaption.

In addition, recovery of other elevated CYP levels was shown, which indicates reconstitution of the hepatic pharmacological response. Our results of reconstituted levels of Cyp1a2 underline previously detected elevated levels of cytochrome P450 1A2 during flight compared to post-flight [22], showing the importance to monitor liver performance during flights. Numerous drugs are turned over in the liver by CYP1A2 [23] illustrating its immense clinical importance. However, we should consider that the observed reconstitution of protein levels could be a consequence of stress during biosatellite re-entry and the landing procedure. To address this concern, mice could pass 30 days on a manned space station with following euthanization on the space station, thus, avoiding stressful landing conditions. This approach could also elucidate direct effects of microgravity or radiation on the liver by excluding stressful landing as a source for proteomic changes, which cannot be excluded from our study.

With relation to energy metabolism, up-regulation in proteins involved in amino acid metabolic processes in the re-adaptation group may indicate an increased level of gluconeogenesis. Initiation of gluconeogenesis is started during lack of cellular glucose caused by short-term fasting [7]. Therefore, prolonged spaceflights may result in reduced glucose uptake during assimilation to Earth conditions. We also assume restoration of bile acid secretion seven days after landing due to restored levels of transmembrane proteins related to bile acid secretion.

In this study, we did not perform quantitative validation using a targeted mass-spectrometry technique. However, Cyp1a1 and Cyp2d9 up-regulation, as well as Cyp3a11 down-regulation is consistent with our previous data on CYP450 absolute abundance obtained by selected reaction monitoring (SRM) with isotopically labeled peptide standards on the same animals (mice from flight, post-flight, and ground control group) [22]. Label-free quantification is a promising approach due to low-cost and using of high-resolution mass-spectrometers, which yields high precise measurements of several peptides per protein (at least three unique peptides in our study). Shao et al. successfully used labeled-free platform combines shotgun mass-spectrometry, targeted SRM, and computational method (“Standard curve slope”) to identify sex-dependent CYP450 expression patterns. Relative expression of Cyp1a1, Cyp2d10, and Cyp2d26 was analyzed in male and female rat liver microsomes [24].

## 4. Materials and Methods

### 4.1. Samples

This study was approved by IACUC of MSU Institute of Mitoengineering (Protocol No-35, 1 November 2012) and of Biomedical Ethics Commission of IBMP (protocol No-319, 4 April 2013) and performed in accordance to the European Convention for the Protection of Vertebrate Animals used for Experimental and Other Scientific Purposes. Male C57/BL6 mice (four–five months old) were selected for spaceflight and ground control experiments. At the time of spaceflight and at the start of the related ground control experiments, the mice had average weight 22 ± 2 g. All mice were pathogen-free. To avoid violent behavior mice were trained before the flight and ground control experiment to form the stable groups of three mice each. Animals were adapted to paste diet. Stable cohorts of three mice were housed in each habitat during spaceflight and ground control experiment. During spaceflight the mice were fed with paste-like food with 76–78% water content developed at IBMP RAS. The mice were kept in a natural light-dark cycle (12 h light and 12 h dark). For more details about experimental animals see Andreev-Andrievsky et al. [1]. In our experiment, we compared liver proteomes of the mice which passed 30 days (from 19 April 2013 to 19 May 2013) in a biosatellite in space and sampled 13–25 h after landing (flight group) with mice of the same spaceflight sampled seven days after landing (post-flight group). Mice held in a biosatellite on Earth in the similar cage from 26 July 2013 to 26 August 2013 under the corresponding housing and climate conditions (temperature, humidity, and atmosphere gas composition) served as control group. The environmental conditions as well as food delivery were continuously recorded during spaceflight. All mice were euthanized by cervical dislocation; livers were immediately sampled and stored at −80 °C until further procedures. 

### 4.2. Sample Preparation

Twenty milligrams of each mouse liver were washed with PBS, homogenized in 200 μL lysis buffer (4% SDS in 0.1 M Tris-HCl pH 8.5) and centrifuged at 3000× *g* at 4 °C for 5 min. Total protein content was measured according to the BCA method [25]. A total protein amount of 100 μg for each sample was used for tryptic digestion according to the common FASP protocol [26]. Briefly, detergents in the samples were exchanged with 100 mM Tris-HCl (pH 8.5) using Microcon filters (10 kDa cut off, Millipore, Bedford, MA, USA). Protein disulfide bridges were reduced with 100 mM 1,4-dithiothreitol in 100 mM Tris-HCl (pH 8.5), alkylation of thiols was performed with 55 mM iodacetamide in 8 M urea/100 mM Tris-HCl (pH 8.5). Tryptic digestion with a trypsin (Sequencing Grade Modified, Promega, Madison, WI, USA) to protein ratio of 1:100 was carried out overnight at 37 °C in a 50 mM tetraethylammonium bicarbonate (pH 8.5) followed by an additional digestion step under the same conditions with 2 h duration.

### 4.3. LC-MS/MS

We separated 2 μg peptides for each sample with high-performance liquid chromatography (HPLC, Ultimate 3000 Nano LC System, Thermo Scientific, Rockwell, IL, USA) in a 15-cm long C18 column with an inner diameter of 75 μm (Acclaim^®^ PepMap™ RSLC, Thermo Fisher Scientific, Rockwell, IL, USA). The peptides were eluted with a gradient from 5–35% buffer B (80% acetonitrile, 0.1% formic acid) over 115 min at a flow rate of 0.3 μL^−1^ min. Total run time including 90 min to reach 99% buffer B, flushing 10 min with 99% buffer B and 15 min re-equilibration to buffer A (0.1% formic acid) amounted 155 min. Further analysis was performed with a Q Exactive HF mass spectrometer (Q ExactiveTM HF Hybrid Quadrupole-OrbitrapTM Mass spectrometer, Thermo Fisher Scientific, Rockwell, IL, USA). Mass spectra were acquired at a resolution of 60,000 (MS) and 15,000 (MS/MS) in a *m/z* range of 400−1500 (MS) and 200–2000 (MS/MS). An isolation threshold of 100,000 counts was determined for precursor’s selection and up to top 25 precursors were chosen for fragmentation with high-energy collisional dissociation (HCD) at 25 NCE and 100 ms accumulation time. Precursors with a charged state of +1 were rejected and all measured precursors were excluded from measurement for 20 s. At least three technical runs were measured for each sample. The mass spectrometry proteomics data have been deposited to the ProteomeXchange Consortium (http://proteomecentral.proteomexchange.org) via the PRIDE partner repository [27] with the dataset identifier PXD005102.

### 4.4. Data Processing

The obtained raw data were processed using the MaxQuant software [28] (version 1.5.5.1, Jürgen Cox, Max Planck Institute of Biochemistry, Martinsried, Germany) with the built-in search engine Andromeda [29]. Protein sequences of the complete mouse proteome provided by Uniprot (August 2016) was used for protein identification with Andromeda. Carbamidomethylation of cysteines was set as fixed modification and protein N-terminal acetylation as well as oxidation of methionines was set as variable modification for the peptide search. A maximum mass deviation of 4.5 ppm was allowed for precursor’s identification and 20 ppm were set as match tolerance for fragment identification (acquisition in Orbitrap). Up to two missed cleavages were allowed for trypsin digestion. The software option “Match between runs” was enabled and features within a time window of 0.7 min were used to match between runs. The false discovery rates (FDR) for peptide and protein identifications were set to 1%. Only unique peptides were used for label-free quantification (LFQ) according to the method described by Cox et al. [30].

### 4.5. Statistical Analysis

The obtained LFQ intensities were filtered and statistically analyzed using the Perseus environment. Protein groups identified only by peptides with modified sites, contaminant matches and matches to the reverse database were removed. Proteins identified by at least two unique peptides were determined as identified, whereas only proteins identified with three unique peptides were used for quantification. Solely protein groups that were at least once quantified in each group were included in our observations. Normalization was performed by built-in MaxQuant algorithm [30]. The LFQ intensities were transformed by log2(x) and missing LFQ intensity values (NaN) were replaced using low LFQ intensity values from the normal distribution (width = 0.3, down shift = 1.8). Unpaired Student’s *t*-tests were used to compare two groups, whereas the ANOVA test was used for multiple-sample testing. To introduce an artificial variance for small variance values, the constant S0 was set to 0.1 for all statistical tests. A permutation-based FDR of 5% was used for truncation of all tests results. Additionally, only proteins with a probability for significant protein abundance changes with a *p*-value <0.05 were used for fold change visualization in the presented tables.

## 5. Conclusions

Studying the alterations of protein level induced by spaceflights in model organisms such as mice revealed proteome changes suggesting impaired fatty acid oxidation leading to lipotoxicity, as well as altered glucose up-take and bile secretion. These results offer the opportunity to adjust the drug treatment and nutrition for future long-termed spaceflights of humans and the duration of medical monitoring after spaceflights.

## Figures and Tables

**Table 1:** Top 10 annotation terms with the highest FDR in enrichment analysis of protein clusters A and B from unsupervised hierarchical clustering. (GOCC = GeneOntology cell compartment; KEGG = Kyoto Encyclopedia of Genes and Genomes; GOBP = GeneOntology biological process; GOMF = GeneOntology molecular function.)

Type	Name	*p*-Value	Enrichment	Total	In Cluster	Cluster Size	Benj. Hoch. FDR
**Cluster A: Significantly Higher Protein Levels in Post-flight Group**							
GOCC name	Ribonucleoprotein complex	1.62 × 10^−14^	1.92	55	52	218	1.90 × 10^−12^
KEGG name	Ribosome	1.70 × 10^−11^	1.97	38	37	218	3.13 × 10^−9^
GOBP name	Cellular amino acid metabolic process	2.23 × 10^−12^	1.82	59	53	218	7.79 × 10^−9^
GOBP name	Cellular nitrogen compound metabolic process	1.07 × 10^−11^	1.54	116	88	218	1.87 × 10^−8^
GOMF name	Structural constituent of ribosome	3.68 × 10^−11^	1.97	37	36	218	3.37 × 10^−8^
GOBP name	Nitrogen compound metabolic process	3.29 × 10^−11^	1.52	119	89	218	3.83 × 10^−8^
GOCC name	Mitochondrial matrix	1.69 × 10^−9^	1.91	36	34	218	9.86 × 10^−8^
GOBP name	Cellular amine metabolic process	6.56 × 10^−10^	1.71	63	53	218	5.73 × 10^−7^
GOBP name	Translation	1.69 × 10^−9^	1.91	36	34	218	9.81 × 10^−7^
GOBP name	Macromolecule biosynthetic process	1.41 × 10^−9^	1.76	53	46	218	9.84 × 10^−7^
**Cluster B: Significantly Lower Protein Levels in Post-flight Group**							
GOCC name	Membrane part	1.04 × 10^−30^	1.65	173	145	224	4.85 × 10^−28^
GOCC name	Intrinsic to membrane	6.62 × 10^−23^	1.80	102	93	224	1.03 × 10^−20^
GOCC name	Integral to membrane	6.62 × 10^−23^	1.80	102	93	224	1.55 × 10^−20^
GOCC name	Organelle membrane	3.49 × 10^−14^	1.47	157	117	224	3.26 × 10^−12^
GOCC name	Endoplasmic reticulum part	7.21 × 10^−12^	1.72	69	60	224	5.63 × 10^−10^
GOCC name	Endoplasmic reticulum	5.52 × 10^−11^	1.84	46	43	224	3.69 × 10^−9^
GOCC name	Endoplasmic reticulum membrane	2.47 × 10^−9^	1.72	54	47	224	1.28 × 10^−7^
GOCC name	Plasma membrane part	5.85 × 10^−8^	1.67	52	44	224	2.74 × 10^−6^
GOCC name	Membrane	4.95 × 10^−7^	1.19	260	157	224	2.10 × 10^−5^
GOMF name	Transporter activity	1.46 × 10^−6^	1.78	31	28	224	2.67 × 10^−4^

**Table 2:** Fold-change of proteins annotated against KEGG database.

Protein Name	Uniprot ID	Fold Change: Control/Post-flight	Fold Change: Flight/Post-flight	Student’s *t*-Test *q*-Value	ANOVA *q*-Value
**Bile Secretion^1^**					
Solute carrier organic anion transporter family member 1B2	Q9JJL3	-	1.7	0.0187	0.0143
ATP-binding cassette sub-family G member 2	Q7TMS5	-	2.7	0.0106	0.0131
Scavenger receptor class B member 1	Q4FK30	*		>0.05	0.0198
Solute carrier family 22 member 1	O08966	*		>0.05	0.0197
Sodium/potassium-transporting ATPase subunit β-1	Q545P0	-	1.6	0.0127	0.0132
Sodium/potassium-transporting ATPase subunit beta-3	Q544Q7	-	2.4	0.0171	0.0116
Aquaporin-1	Q02013	*		>0.05	0.0211
Solute carrier organic anion transporter family member 1A1	Q53ZW9	-	4.0	0.0293	0.0291
Sodium/potassium-transporting ATPase subunit alpha-1	Q8VDN2	1.9	1.6	0.0165	0.0122
Epoxide hydrolase 1	Q9D379	-	1.9	0.0077	0.0278
**PPAR Signaling Pathway ^1^**					
Long-chain specific acyl-CoA dehydrogenase, mitochondrial	P51174	-	0.6	0.0340	0.0374
Long-chain-fatty-acid--CoA ligase 1	D3Z041	-	1.9	0.0052	0.0075
Very long-chain acyl-CoA synthetase	O35488	-	1.5	0.0247	0.0341
Cytochrome P450 4A10	O88833	*		>0.05	0.0154
Fatty acid-binding protein, liver	Q3V2F7	-	3.9	0.0080	0.0187
Acyl-CoA-binding protein	Q548W7	*		>0.05	0.0374
Medium-chain specific acyl-CoA dehydrogenase, mitochondrial	P45952	0.5	0.6	0.0457	0.0132
Carnitine O-palmitoyltransferase 2, mitochondrial	P52825	-	1.7	0.0110	0.0115
Carnitine O-palmitoyltransferase 1, liver isoform	Q7TQD5	*		>0.05	0.0153
CD36 antigen, isoform CRA_a	Q3UAI3	-	2.1	0.0044	0.0255
Cytochrome P450 4A12A	Q91WL5	-	5.0	0.0113	0.0128
Peroxisomal bifunctional enzyme	Q9DBM2	-	0.6	0.0141	0.0116
Phosphoenol pyruvate carboxy kinase, cytosolic [GTP]	Q9Z2V4	*		>0.05	0.0271
**Retinol Metabolism ^1^**					
Cytochrome P450 1A2	B6VGH4	-	2.7	0.0117	0.0125
Cytochrome P450 2B10	Q9WUD0	*		>0.05	0.0120
Cytochrome P450 4A10	O88833	*		>0.05	0.0154
Cytochrome P450 3A11	Q3UEN8	-	0.7	0.0198	0.0166
UDP-glucuronosyltransferase	Q3UEP4	-	2.1	0.0167	0.0132
Cytochrome P450 3A13	Q3UW87	-	0.4	0.0121	0.0279
UDP-glucuronosyltransferase 1-1	Q63886	-	1.2	0.0420	0.0185
UDP-glucuronosyltransferase 1-6	Q64435	-	2.0	0.0179	0.0410
Cytochrome P450 2C54	Q6XVG2	*		>0.05	0.0476
UDP-glucuronosyltransferase 2A3	Q8BWQ1	-	1.9	0.0109	0.0132
UDP-glucuronosyltransferase	Q8K154	-	1.9	0.0172	0.0126
UDP-glucuronosyltransferase	Q8R084	-	3.5	0.0118	0.0119
Cytochrome P450 2C70	Q91W64	*		>0.05	0.0340
Cytochrome P450 4A12A	Q91WL5	-	5.0	0.0113	0.0128
Dehydrogenase/reductase SDR family member 4	Q99LB2	-	1.7	0.0108	0.0131
**Cytochromes ^2^**					
Cytochrome P450 2D26	Q8CIM7	1.5	-	0.0474	0.0120
Cytochrome P450 4V3	B2RSR0	-	2.3	0.0175	0.0176
Cytochrome P450 1A2	B6VGH4	-	2.7	0.0117	0.0125
Cytochrome P450 2B10	Q9WUD0	*		>0.05	0.0120
Cytochrome P450 4A10	O88833	*		>0.05	0.0154
Cytochrome P450 2D9	P11714	-	6.1	0.0117	0.0106
Cytochrome P450 2D10	P24456	-	2.1	0.0134	0.0152
Cytochrome P450 2F2	P33267	-	1.6	0.0264	0.0198
Cytochrome P450 3A11	Q3UEN8	-	0.7	0.0198	0.0166
Cytochrome P450 3A13	Q3UW87	-	0.4	0.0121	0.0279
Cytochrome P450 2C54	Q6XVG2	*		>0.05	0.0476
Cytochrome P450 2C70	Q91W64	*		>0.05	0.0340
Cytochrome P450 4A12A	Q91WL5	-	5.0	0.0113	0.0128

* Proteins with ANOVA-significant but two-sample *t*-test (Student’s *t*-test) insignificant *q*-value; ^1^ Proteins derived from partially same KEGG names (Kyoto Encyclopedia of Genes and Genomes); ^2^ Members of the Cytochrome P450 superfamily with significant protein abundance changes.

## Supplementary Materials

Supplementary materials can be found at www.mdpi.com/1422-0067/18/8/1763/s1.

## Abbreviations

PPAR	Peroxisome proliferator-activated receptor
FDR	False discovery rates
CYP	Cytochrome P450
FASP	Filter aided sample preparation
LFQ	Label-free quantification
NAFLD	Non-alcoholic fatty liver disease
FABP	Fatty acid binding protein
ID	Identification number
GOCC	GeneOntology cell compartment
GOMF	GeneOntology molecular function
GOBP	GeneOntology biological process
KEGG	Kyoto Encyclopedia of Genes and Genomes
