# Supplementary material for: Re-Adaption on Earth after Spaceflights Affects the Mouse Liver Proteome
Source: Int J Mol Sci. 2017 Aug 12;18(8):1763. doi: 10.3390/ijms18081763 (PMC5578152; doi:10.3390/ijms18081763)
Supplement: Supplementary file 1 [file ijms-18-01763-s001.zip › Figure S1.pdf]

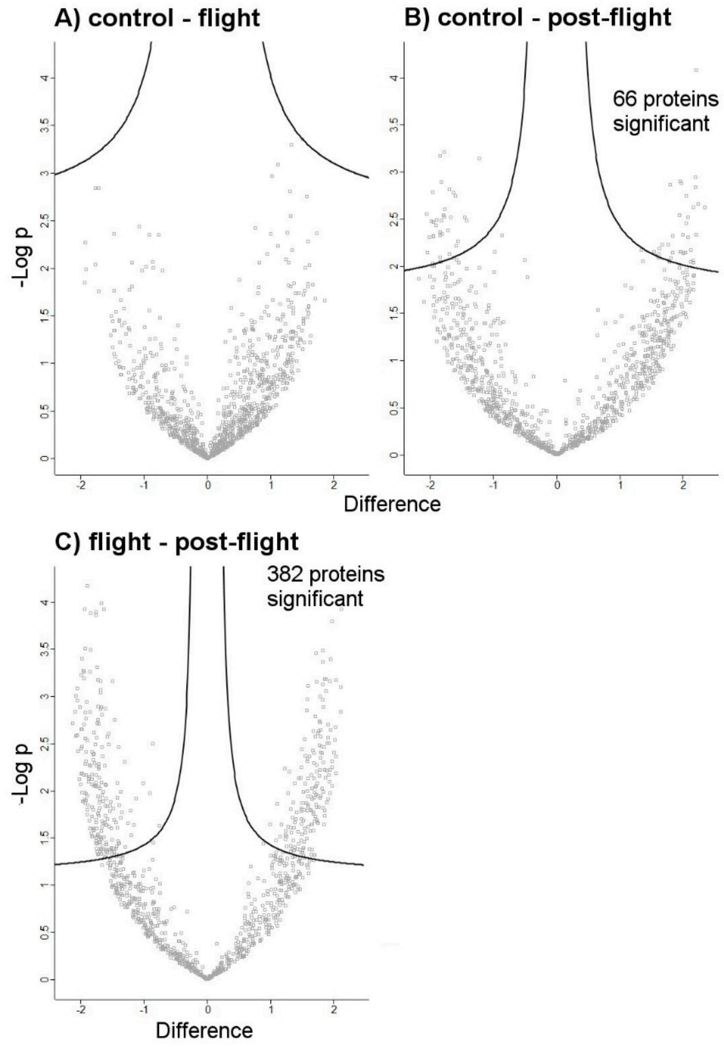

Figure S1. Comparison of significant changes in the protein expression level between flight, post-flight, and control group (Student's t-test, truncation: permutation-based FDR=0.05, S0=0.1). Normalized data of LFQ intensities were used for visualization (Z-score) of differences in protein level. All quantified proteins were marked as gray squares. Significantly changed proteins between two conditions appeared above the line. (A) No observed significant change in protein abundance between control and flight group (B) Comparison of the conditions control and post-flight. (C) Comparison of the conditions flight and post-flight.
