# Supplementary material for: Re-Adaption on Earth after Spaceflights Affects the Mouse Liver Proteome
Source: Int J Mol Sci. 2017 Aug 12;18(8):1763. doi: 10.3390/ijms18081763 (PMC5578152; doi:10.3390/ijms18081763)
Supplement: Supplementary file 1 [file ijms-18-01763-s001.zip › Supplementary Overview.pdf]

Supplementary Overview

Table S1: List of all identified proteins based on at least two unique peptides for each protein.
Table S2: List of all quantified proteins based on at least three unique peptides for each protein. Overview of relative protein intensities (LFQ intensities) provided by MaxQuant.
Table S3: Lists of proteins with significantly changed abundance from multiple sample test (ANOVA model, artificial background constant S0=0.1, permutation-based FDR of 5% for truncation).
Table S4: Enrichment analysis and the corresponding protein members based on unsupervised hierarchical clustering in Figure 2.
Table S5: Lists of proteins with significantly changed abundance confirmed by Student's t-tests using a permutation-based FDR of 5% for truncation.
Protein level change in: control versus post-flight
flight versus post-flight
only significant in control versus post-flight
common: significant change observed in both comparisons
LFQ intensities of proteins with specific KEGG annotations
Figure S1. Comparison of significant changes in the protein expression level between flight, post-flight, and control group (Student's t-test, truncation: permutation-based FDR=0.05, S0=0.1).
